# A novel application of process mapping in a criminal justice setting to examine implementation of peer support for veterans leaving incarceration

**DOI:** 10.1186/s40352-019-0085-x

**Published:** 2019-03-26

**Authors:** Bo Kim, Megan B. McCullough, Molly M. Simmons, Rendelle E. Bolton, Justeen Hyde, Mari-Lynn Drainoni, B. Graeme Fincke, D. Keith McInnes

**Affiliations:** 1 VA Center for Healthcare Organization and Implementation Research, Bedford/Boston, MA USA; 2 Harvard Medical School, Boston, MA USA; 3 Boston University School of Public Health, Boston, MA USA; 4 RAND Corporation, Boston, MA USA; 5 Brandeis University Heller School for Social Policy and Management, Waltham, MA USA; 6 Boston University School of Medicine, Boston, MA USA

**Keywords:** Process mapping, Qualitative analysis, Implementation methods, Intervention design, Peer support, Care coordination, Community providers, Incarceration

## Abstract

**Background:**

Between 12,000 and 16,000 veterans leave incarceration every year, yet resources are limited for reentry support that helps veterans remain connected to VA and community health care and services after leaving incarceration. Homelessness and criminal justice recidivism may result when such follow-up and support are lacking. In order to determine where gaps exist in current reentry support efforts, we developed a novel methodological adaptation of process mapping (a visualization technique being increasingly used in health care to identify gaps in services and linkages) in the context of a larger implementation study of a peer-support intervention to link veterans to health-related services after incarceration (https://clinicaltrials.gov/, NCT02964897, registered November 4, 2016) to support their reentry into the community.

**Methods:**

We employed process mapping to analyze qualitative interviews with staff from organizations providing reentry support. Interview data were used to generate process maps specifying the sequence of events and the multiple parties that connect veterans to post-incarceration services. Process maps were then analyzed for uncertainties, gaps, and bottlenecks.

**Results:**

We found that reentry programs lack systematic means of identifying soon-to-be released veterans who may become their clients; veterans in prisons/jails, and recently released, lack information about reentry supports and how to access them; and veterans’ whereabouts between their release and their health care appointments are often unknown to reentry and health care teams. These system-level shortcomings informed our intervention development and implementation planning of peer-support services for veterans’ reentry.

**Conclusions:**

Systematic information sharing that is inherent to process mapping makes more transparent the research needed, helping to engage participants and operational partners who are critical for successful implementation of interventions to improve reentry support for veterans leaving incarceration. Even beyond our immediate study, process mapping based on qualitative interview data enables visualization of data that is useful for 1) verifying the research team’s interpretation of interviewee’s accounts, 2) specifying the events that occur within processes that the implementation is targeting (identifying knowledge gaps and inefficiencies), and 3) articulating and tracking the pre- to post-implementation changes clearly to support dissemination of evidence-based health care practices for justice-involved populations.

**Electronic supplementary material:**

The online version of this article (10.1186/s40352-019-0085-x) contains supplementary material, which is available to authorized users.

## Background

Reentry into the community, of individuals who have been incarcerated, is a complex process. It is comprised of multiple interdependent steps, which involve a collection of resources, services, and organizations that interact with individuals leaving incarceration and undergoing reentry. A shared understanding of this process, among all involved individuals and teams, is thus critical in implementing an intervention to change and improve the process. We describe here an experimental use of process mapping to better enable such shared understanding of the process, for efficient and collaborative implementation work to enhance support for veterans who are leaving incarceration and reentering the community.

**Fig. 1:**
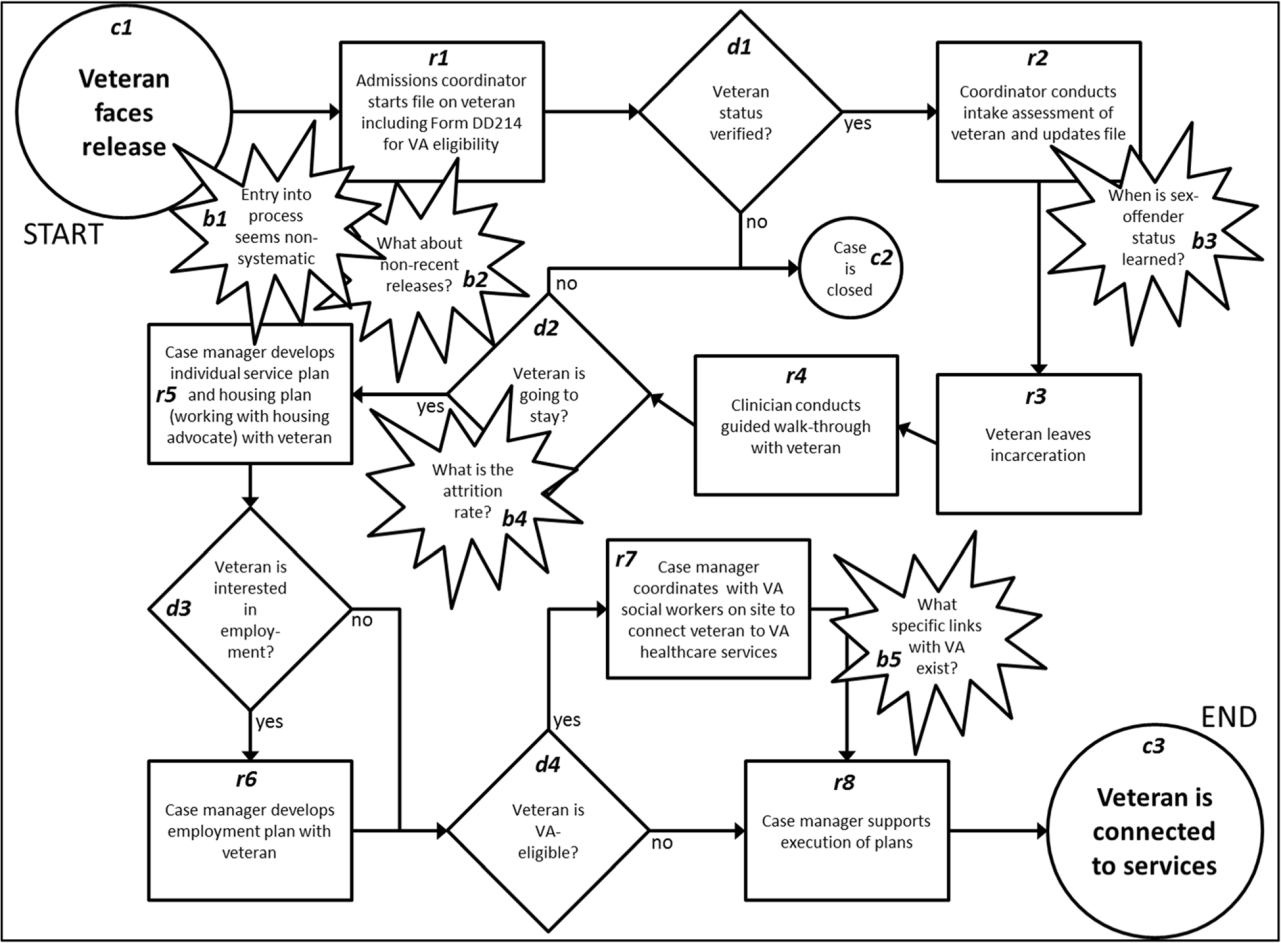
Example of an interview-based process map generated for this study. The start and end points that were used to bound the discussion are indicated as *circles* (numbered with letter “c”) at the top left and bottom right corners of the map, respectively, and *arrows* between the shapes mark the sequence in which individual events (represented as *rectangles* on the map, numbered with letter “r”) take place within the process. A *diamond* (numbered with letter “d”) is used when there is a decision point in the process, where events following the point are determined by the answer to the question asked within the diamond. *“Burst” shapes* (numbered with letter “b”) indicate uncertainties, gaps, bottlenecks, or inefficiencies. Notice the small circle (above and to the right of the center of the figure, labeled “c2”) that demonstrates an alternative end point to the process based on the answer to a decision point question (e.g., in this case, whether veteran status can be verified). Form DD214 (in the rectangle labeled “r1”) is documentation by the United States government on individuals leaving military service, used to verify their veteran status and eligibility for receiving VA services

Process mapping is a visualization technique widely used in manufacturing and business, and more recently applied to health care and social services (Gimbel et al. 2014; Zelenitsky et al. 2014). It contributes to precise descriptions of complex processes and illustrates where steps in a process may diverge, occur in parallel, or exhibit gaps and uncertainties. The health care domain in which process maps are most often used is quality improvement, where it is deemed essential to first assess the shortcomings of the current process then design a new one to specifically target those very shortcomings. The common steps included in process mapping, as outlined within the Institute for Healthcare Improvement’s QI Essentials Toolkit (QI Essentials, 2017), are to (i) gather information about the process from those who know it the best, (ii) define clearly the first and last step in the process, (iii) articulate each step in the process (as it actually is, not as it should be), and (iv) represent using visual shapes (e.g., circles, rectangles, arrows; Fig. 1’s legend provides more detail on each shape) the articulated process.

What is yet to be explored is whether process mapping can be used with formative assessment (Stetler et al. 2006) data (e.g., qualitative stakeholder interview data), prior to any interventions being developed. It will be valuable to determine if process mapping helps to understand the spatiotemporal connections between the steps of a given process, which can in turn be used to identify enhancements or modifications to programs, services, and other interventions that often involve multiple interrelated stakeholders (i.e., individuals impacting and/or impacted by the process) and organizations. The reentry support process that we focus on here exemplifies one such complex process, which involves multiple steps, institutions (federal, state, city, and private), and contingencies (e.g., the type of criminal offense often dictates available paths and resources), and it may unfold over days or weeks. Thus, using process mapping, our aims were (i) to understand the current process through which veterans are supported by organizations that work to connect them to health care and other services after leaving incarceration (“reentry support organizations”) and (ii) to identify where in the current reentry support process there are challenges that can potentially be addressed through the addition of peer support specialists.

This work was a formative step in a larger Post-Incarceration Engagement (PIE) project to implement a peer-based intervention to enhance reentry support for veterans (Simmons et al. 2017). Between 12,000 and 16,000 veterans leave incarceration every year (Homeless Services Cube 2014). Based on studies not limited to the veteran population, individuals leaving incarceration are known to often face discrimination or exclusion upon reentering their communities (Dennis et al. 2015), and homelessness, relapse in substance use behaviors, and increased mental health problems are a few of many issues related to such vulnerable populations facing discrimination and exclusion (Campbell et al. 2015; Mays et al. 2017). About 50% of veterans incarcerated in state prisons report having experienced symptoms of mental health disorders, and about 75% report using drugs prior to incarceration (Noonan and Mumola 2007). The period of transition out of incarceration is a particularly vulnerable time for these veterans, as they are likely to experience a disruption in their established mental health and substance use treatment and associated medications (Baillargeon et al. 2009; Meyer et al. 2011; Massoglia and Schnittker 2009; Hartwell et al. 2013).

The Department of Veterans Affairs (VA)’s Health Care for Reentry Veterans program links them to VA and community health care services (VHA Health Care for Reentry Veterans 2014). However, resources are limited and many veterans may not receive sufficient post-release emotional and instrumental support (Wortzel et al. 2012). Homelessness and criminal justice recidivism may result when such follow-up and support are lacking (Baillargeon et al. 2009; Meyer et al. 2011; Swan 2015). The main aims for the larger PIE project are therefore to (i) conduct contextual analysis to identify VA and community reentry resources, and describe how reentry veterans use them (this paper falls under this first aim of the larger project) and (ii) implement peer-support, in one state, to link reentry veterans to VA primary care, mental health, and substance use disorder services, then (iii) port the peer-support intervention to another, geographically and contextually different state (Simmons et al. 2017).

Peer programs have been found to decrease risk behaviors and improve health among justice-involved populations (Bagnall et al. 2015; Nyamathi et al. 2015). Benefits of using peers over non-peer professionals include how peers have had more similar experiences as support recipients, and are thus able to offer relevant advice and hope (Solomon et al. 1998; Blodgett et al. 2013). Especially for individuals with mental health and substance use disorders, peer programs have demonstrated effectiveness in improving their linkage and engagement with services (Bagnall et al. 2015; Nyamathi et al. 2015; Blodgett et al. 2013). Although there is some evidence that peer programs may improve a variety of outcomes for the reentry population (Rowe et al. 2007; Goldstein et al. 2009; Marlow et al. 2015), there are few published interventions focusing on veterans. To address this evidence gap, this paper describes how process mapping contributed to the design of a locally relevant peer-support intervention to assist veterans as they return to community settings after incarceration. Process mapping was used to extract and analyze process-related information that is available within formative qualitative interview data. This work can be understood in the context of the Sequential Intercept Model (SIM), which outlines the criminal justice continuum from arrest, to court appearances, and to community reintegration (Munetz and Griffin 2006). But whereas SIM is a generalized outline of the entire criminal justice system indicating points for potentially diverting individuals out of the system, our examination takes a micro view of the last two steps in SIM – release from incarceration and support in the community.

We demonstrate how we first gathered information from qualitative interviews with representatives of reentry support organizations. We share how we systematically generated process maps based on the information gathered, and we provide examples of the maps themselves to help illustrate their utility. Some data are provided in this paper (process details from the interviews), but our focus is on how process mapping can inform intervention planning when interventions are to be implemented within contexts that involve multiple stakeholders and organizations. Details of the larger project that are less directly related to this process mapping work (e.g., procedure for implementing and evaluating the peer support intervention, grounded thematic analysis using interview data on topics beyond the reentry support process) are provided within the larger study’s protocol paper (Simmons et al. 2017).

Through this work, we portray several reasons why other researchers should consider process mapping to guide intervention planning and implementation for studies of justice-involved populations. First, we show that process mapping can be conducted based on formative assessment data from stakeholder interviews, a data collection approach often included in studies of justice-involved populations (Taxman et al. 2013; Shafer et al. 2014; Belenko et al. 2013). Second, the reentry support process that we are targeting is one that involves multiple interrelated stakeholders and organizations (for which process mapping is well suited), which is a common situation faced by many studies of justice-involved populations being carried out (Becan et al. 2018; Fisher et al. 2018; Friedmann et al. 2013). Third, our project will later involve tailoring our peer-support intervention and implementation (first developed for Massachusetts) to other states’ contexts (for which process mapping helps to visualize key process differences across contexts that need to be accounted for), which is similar to expansion efforts of many studies on justice-involved populations that target multiple sites (Ducharme et al. 2013).

## Methods

The study was submitted to the Institutional Review Board at the Edith Nourse Rogers Memorial Veterans Hospital (Bedford, Massachusetts, USA), which reviewed the study then designated the study as a quality improvement project, waiving the study’s need for ethics approval. The larger PIE project, of which this study was a part, was designed to examine the effects of implementing a peer-based reentry support intervention on veteran linkage to and engagement in VA health care (Simmons et al. 2017). This study was one component of the initial formative evaluation efforts of the larger project. As described below, it focused on comprehensively outlining the existent (i.e., pre-intervention) reentry process in one state, using process mapping based on interview data.

### Data collection through qualitative interviews

We conducted semi-structured qualitative interviews with staff from reentry support organizations across Massachusetts. We focused on Massachusetts to have the interviews directly inform the aforementioned PIE project’s efforts to implement peer-based services in Massachusetts to support veterans’ reentry after leaving incarceration (Simmons et al. 2017). These interviews sought descriptions of the reentry process, including planning for a veteran’s reentry prior to release from incarceration, navigating decision points prior to and following release, connecting the veteran to needed services following release, and providing ongoing support (where applicable) to the veteran.

Participants were recruited using a combination of snowball and purposive sampling strategies, in order to help ensure that we gather data on each of federal, state, and community organization perspectives. We began by identifying potential participants at relevant reentry-involved organizations through conversations with VA’s homelessness programs and Health Care for Reentry Veterans (HCRV) staff (Blue-Howells et al. 2013) familiar with available reentry support programs. Additional organizations were identified by interview participants, and we pursued inviting the organizations’ reentry support staff when adding them increased the breadth of representation that we were seeking across a variety of reentry support programs.

We conducted sixteen hour-long interviews between March and September 2016. Five participants were staff members of the Massachusetts Department of Corrections, and eleven participants were employees at community, state, or VA agencies providing reentry support to individuals leaving incarceration. Each participant’s professional role involved contributing to pre-release reentry planning and/or post-release reentry assistance, although their exact organizational positions (e.g., supervisory versus front-line) varied from one another. Verbal informed consent was obtained from all participants. The need for written consent had been waived by the Institutional Review Board at the Edith Nourse Rogers Memorial Veterans Hospital, because the study was deemed a quality improvement project. Interviews were audio-recorded and transcribed verbatim. Recording was not possible with some correctional facility staff, in which case detailed notes were taken by the interviewer(s).

### Data interpretation and analysis through process mapping

We carried out data interpretation and analysis in three phases. First, we reviewed reentry process-related data from all sixteen interviews. Second, we drafted process maps for four of the interviews that collectively provided the richest detail across the federal, state, and community organizations that take part in reentry support. Third, we refined the drafted process maps using confirmatory and additional information from the other twelve interviews, hence ensuring that key reentry process-related information from all interviews are reflected in the process maps. Details of each phase are explained below.

First phase (reviewing reentry process-related data from all sixteen interviews): Five research team members were each assigned as the primary preliminarily reviewer of data from two to four interviews out of the sixteen interviews. They were then asked to create a brief summary for each interview out of the sixteen interviews for which they were the primary reviewer, by filling out a summary document template with designated spaces for (i) background information on the participant, (ii) context of the participant’s organization, and (iii) key points from the reviewed interview content. These key points particularly focused on the level of detail available from the interview on the participant’s knowledge and experience regarding pre-release reentry planning and post-release reentry assistance of veterans leaving incarceration.

Second phase (drafting process maps for four of the interviews that collectively provided the richest detail across the federal, state, and community organizations that take part in reentry support): The team members reviewed each other’s summaries, then purposively and collaboratively selected four out of the sixteen interviews that were deemed to together offer the best available collection of both the breadth and detail on reentry support to inform our process maps. These interviews represented agencies that provided a range of reentry support, including housing, employment assistance, physical and behavioral health services, and case management. Importantly, the selected interviews also provided information about reentry support that crosses boundaries of federal, state, and community organizations – a reality (and complication) of many reentry support efforts. In these interviews, interviewees both described the process of reentry support from their own perspective and reflected on what is working well versus needing improvement.

Five to seven researchers took part in each of the sessions during which an interview-based process map was generated. Specifically, for each of the four interviews, the collaborative generation of a process map based on the interview included the following steps:

1. Each researcher individually reviewed data from the interview that had been conducted with staff from a reentry support organization. Particular attention was paid to information regarding what reentry support events are carried out by whom and in what order. Researchers noted uncertainties about the process that they wanted discussed in the subsequent group meeting.
2. The group meeting was convened of participating researchers who had individually reviewed the interview data. Guided by Bens’ meeting facilitation techniques (Bens 2012) [an interrelated and interactive set of practical strategies for keeping meeting-based discussions productive and relevant – e.g., straightforward tasks applicable to any meeting (e.g., setting ground rules, clarifying meeting objectives), more conceptual guidance on how to lead group meetings that draw on all participants’ perspectives while moving toward consensus (e.g., to finalize a process map), and advanced tactics to be applied in case of conflict resolution], the moderator (first author) led a consensus-building discussion for a duration of approximately 60 to 75 min. The moderator first clarified the meeting’s objective to generate a process map that reflects the researchers’ shared understanding of the reentry support process based on the reviewed interview data, and provided a reminder that the process of interest only involves events that take place between the specified start and end points (when a veteran is facing upcoming release and when the veteran is connected to his/her needed services, respectively). Discussion was prompted with questions of the types:

- What happens next?
- Who does that?
- Is that done for all cases of release-facing veterans, or only for a subset?
- If only for a subset, what are the decision criteria?

Post-it notes and markers were used by the participating researchers to capture in real-time the evolving map of the process on a white board (a photograph of this is included as Additional file 1). Periodically throughout the session and at the end, the moderator reiterated and asked for revisions to what the visual representation of the map was showing, to help ensure that the representation did in fact capture what all participating researchers thought was being captured.

3. The moderator drafted an electronic version of the map based on the outcome of the meeting. The draft was reviewed by all participating researchers and suggestions were incorporated to finalize the consensus-reached process map.

These steps were followed to generate a process map for each of the four selected interviews.

Third phase (refining the drafted process maps using confirmatory and additional information from the other twelve interviews, hence ensuring that key reentry process-related information from all interviews are reflected in the process maps): For each interview other than the selected four, the researcher who had led its summary documentation, together with others on the research team who had reviewed the summary, closely reviewed and revised the generated process maps to ensure that information available from that particular interview regarding the reentry support process is sufficiently represented. Basing our process mapping primarily on four interviews, while accounting for potential additional information from the other interviews, enabled us to generate a collection of useable process maps that can clearly show the main relevant process steps from multiple perspectives, while not focusing too heavily on details specific to any one interview.

The generated process maps provided explicit visualizations of our shared understanding of the reentry support process, which we then analyzed in two steps. First, we examined uncertainties [i.e., variabilities in sequence of steps within the process and/or lacks of systematized steps that link one part of the process to another (van der Horst & Beulens 2002)], gaps [i.e., discrepancies between what the process is intended to look like and what it actually looks like (Heher & Chen 2017)], bottlenecks [i.e., points within the process where there are delays before the next step occurs (Lummus et al. 2006)], and inefficiencies [i.e., steps that are unnecessarily repeated/replicated (Cima et al. 2011)] within the mapped reentry support process to identify potential areas for improvement. We then compared the analyzed maps to uncover trends in improvement areas that are present across all the maps. We also noted the amount of researcher time spent on each step of the process mapping analysis, in order to be able to provide an estimate of the resource cost associated with our process mapping approach – information that is likely to be essential for future studies considering the use of process mapping.

## Results

**Fig. 2:**
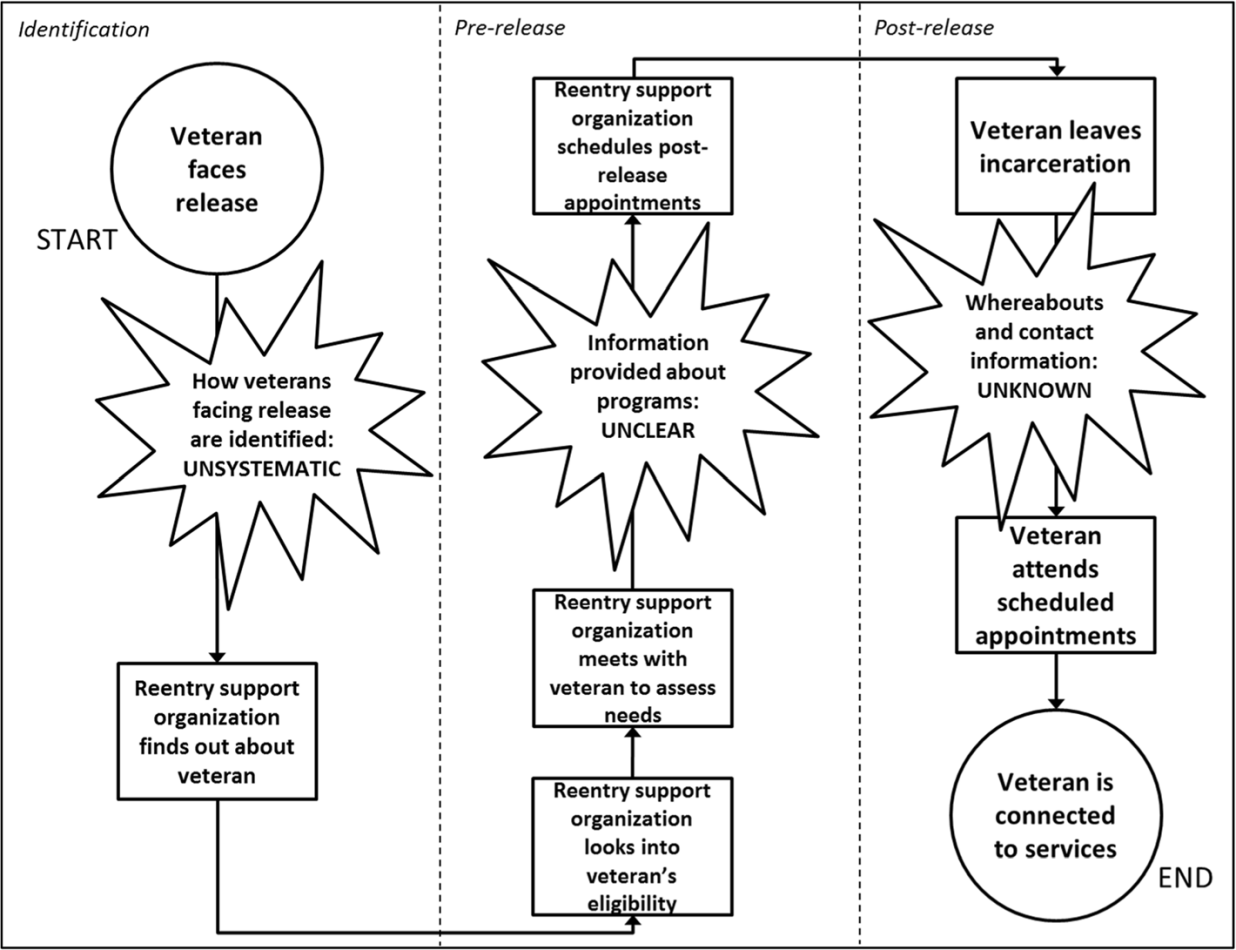
Map of the generalized reentry support process with current challenges at identification, pre-lease, and post-release. This figure is a summarized and generalized process map synthesized from four individual process maps. While the individual maps focus on detailing the process complexities, this generalized map focuses on visualizing major issues that were emphasized repeatedly in the individual maps

Figure 1 provides an illustrative example of a finalized consensus-reached process map based on an interview with staff from a reentry support organization, and Fig. 2 shows the generalized reentry support process of reentry-related events that were common across all the generated process maps.

### Interview-based process map (an illustrative example)

Figure 1’s legend explains the arrows and different shapes on the map – circles, arrows, rectangles, and diamonds. Following the arrows through Fig. 1 allows us to explicitly articulate the steps involved in this organization’s reentry support process, from when there is a veteran being readied for release from incarceration (c1) to when the veteran is connected to the needed services (c3). Several challenges to and uncertainties about the reentry process were brought to light through generating this process map (noted using the burst shapes within the figure). For instance, it was unclear from this particular interview how the organization learns about veteran ex-offenders who were released weeks or months ago but still are in need of services (b2). Also unclear was when in the process sex-offender status is learned (a status that significantly affects housing and other service eligibility), and in turn how that affects the process (b3).

### Generalized reentry support process

Following the creation of consensus-reached process maps based on individual interviews, the second step of our analysis compared the interview-based process maps to identify trends and uncover areas for improvement to target in our intervention. The process maps detailed a challenge for each of the three main periods of the reentry support process – identification, pre-release, and post-release – shown in Fig. 2.

Identification period (left section of Fig. 2): The identification period involves activities that occur during reentry planning for a release-facing veteran when his or her case is being considered by an organization that will assist with supportive services upon the veteran’s release. One interviewee at a service organization said, “[Correctional facilities] all have a reentry specialist, … they know to keep in touch with me about the veteran identified …. Sometimes inmates will write me. … I get a lot of letters.” The process maps indicated that the events through which a reentry-planning organization outside correctional facilities would find out about a release-facing veteran is multi-faceted and not standardized, including generating lists of upcoming releases, receiving letters (e.g., from the probation officer, the social worker, or the veteran), and connecting face-to-face during visits to correctional facilities. We thus found this identification period to be associated with the challenge of systematically identifying release-facing veterans.

Pre-release period (middle section of Fig. 2): The pre-release period involves activities that occur from when a reentry-planning organization outside of correctional facilities finds out about a release-facing veteran through when the organization helps schedule post-release appointments for the veteran to meet identified needs (e.g., health assessment at a primary care clinic, housing appointment, etc.). When asked how veterans might find out about available services and other resources during this period, one interviewee replied that a veteran could receive information during the roughly six-month pre-release period, for example, “… the reentry guide book is in [correctional facilities’] libraries … they then go into a reentry workshop, so the reentry piece kind of covers like available resources and stuff and they talk about the veteran things too.” The process maps demonstrated that there is significant variation in how and what information is made available to release-facing veterans as they go through reentry planning, and that support organizations often are unsure of what reentry information is collectively available to veterans prior to their release. We thus found this pre-release period to be associated with the challenge of informing veterans of available post-incarceration services.

Post-release period (right section of Fig. 2): The post-release period involves activities that occur following a veteran’s release from incarceration. An interviewee shared what is helpful in ensuring that the veteran shows up to services that were arranged for him or her prior to release: “The recovery coach regularly picks people up at prison, from prison and brings them back here [reentry organization’s offices]. … the day of their release. … Otherwise you tend to not see them.” The process maps showed gaps at this stage, common across organizations, related to difficulty maintaining contact with the veteran following his or her release, thwarting attempts to support veterans’ connections to services. Interviewees emphasized the critical need to be able to provide this support immediately upon a veteran’s release, as they consider this time to be particularly influential in deciding the path taken by the veteran as he or she reenters the community. We thus found this post-release period to be associated with the challenge of maintaining contact with veterans.

Figure 2 is a summarized and generalized process map developed from the four individual process maps. As such, it does not detail the complexities that are contained in any of the individual maps, but it enables implementers and intervention developers to focus on major issues that were emphasized repeatedly in the individual maps.

### Researcher time spent on process mapping-based analysis

For each interview analyzed for this study, five to seven participating researchers each spent 90–120 min individually reviewing the data, followed by participation in 60–75 min of group discussion to generate a draft of a process map based on the interview. As the researcher leading this process mapping analysis, the moderator spent an additional 60–75 min putting the draft into electronic form to share with the other researchers. Each participating researcher spent 15–30 min reviewing and revising the electronic draft towards finalization (e.g., Fig. 1). After repeating the above steps for each of the four considered interviews, each researcher spent an additional 15–30 min to examine and articulate trends noticed across all of the individual process maps. The moderator then spent approximately 60 min to synthesize the research team’s noticed trends and visualize them within the generalized process map (Fig. 2).

## Discussion

The interviewed staff from correctional and other agencies shared their contributions to different parts of the overall reentry process, which is experienced from start to end by a veteran leaving incarceration (e.g., correctional facility staff are primarily involved until a veteran’s release from incarceration, other agencies become more involved as a veteran faces release in the upcoming months and they remain involved post-release). Our work was to bring together the information on the different sub-processes identified by the interviewees, to characterize the overall reentry process as experienced by a veteran leaving incarceration.

### The case of our peer-based reentry support project

Process mapping helped bring to light the three main challenges faced regarding (a) systematic identification of release-facing veterans, (b) informing veterans of available services pre-release, and (c) maintaining contact with veterans post-release. Identification of these challenges enabled us to plan for addressing each of them directly as a part of our intervention to incorporate peer specialists into the reentry support process, as well as for defining the specifics of our facilitation strategy for implementing the intervention. Table 1 summarizes our plan, including potential interventional components to address the identified challenges.

**Table 1:** Reentry challenges identified and their associated intervention plans

Period of reentry support	Main challenge identified	Intervention plan to address challenge	Facilitation focus to operationalize intervention plan
Identification	Systematic identification of release-facing veterans	Peers will communicate regularly with correctional facilities’ staff who lead their internal reentry planning efforts	Involve correctional facilities’ staff early on in intervention development to reflect their perspectives in defining peers’ roles, and create relationship-building opportunities between new peers and correctional facilities’ staff
Pre-release	Informing veterans of available service pre-release	Peers, in their meetings with veterans in correctional facilities, will discuss available resources and options Peers will have access to a resource guide, and be trained in how to identify, in the community to which a veteran is returning, additional programs and services such as for health care, housing, and employment	Gather (and define a procedure for maintaining up to date) information on available resources and options from multiple sources that peers can build on Develop a training manual for peers, together with peers’ supervisors and with input from key stakeholder representatives, which will serve as the main step-by-step guide to orienting new peers to serving in the role of assisting with veterans’ reentry
Post-release	Maintaining contact with veterans post-release	Peers will not only note where a veteran is going immediately upon release, but also collect from the veteran contact information for several people who will always know how to reach the veteran	Set up a standardized day-of-release protocol, together with correctional facilities’ staff, for peers to collect the needed contact and other veteran-specific information for maintaining in touch with the veteran following release

### Applicability of the process mapping approach to other projects

Systematic re-application of the demonstrated process mapping approach can help studies better articulate and less ambiguously compare the processes that exist under different contexts within which they plan to implement their intervention, and can also allow for identification of implementation strategies that may work best within each context. Framed in the context of the SIM (Munetz and Griffin 2006), the process maps for our project provide detailed visualizations of where there currently are gaps and challenges within the processes that belong to the SIM’s community reintegration component (covered by the model’s Reentry and Community Support intercepts). Our approach serves as a tool for researchers documenting their studies of transition pathways from prison to community, as recommended by Visher and Travis in their 2003 review (Visher and Travis 2003). Accordingly, with regards to SIM, we would encourage initiatives that focus on other components of SIM to consider the application of this process mapping approach to those other components. For example, a person’s initial detention and court hearings, one of SIM’s intercepts, also can involve multiple agencies, organizations, and jurisdictions, and thus would likely benefit from systematic mapping of the common pathways and services (and also the uncertainties, gaps, and inefficiencies represented by “bursts” in process maps) involved in supporting the justice-involved individual.

We also hope that the estimates of researcher time that was spent on the process mapping-based analysis for our work (provided under Results) can serve as a guide for future studies that consider using process mapping. Additionally collected time data from those studies can then lead to more detailed understanding of the resource cost associated with our proposed process mapping approach, allowing us to compare the cost against other alternative approaches to analyzing formative assessment data for intervention designs and implementation plans involving complex processes.

### Limitations

A limitation of our study is its having generated the process maps based primarily on only a subset of the conducted interviews. We focused process mapping on the purposively selected four interviews in order to account for both breadth and detail regarding reentry support within the process maps while allocating a feasible amount of researcher time to the effort. For each of the other interviews, the researcher leading its summary documentation, together with others on the research team who reviewed the summary, were responsible for ensuring that unique and potentially essential information from it about reentry support steps are still appropriately reflected in our process maps using visualized decisions points and alternative pathways. We would encourage future studies considering process mapping to similarly both account for feasibility and incorporate countermeasures against potential information loss. Our study is also limited in its geographic reach, but given the legal, regulatory, and resource differences between states, it is unlikely that a universal peer-based intervention would be effective across all geographic regions. Process mapping is a method that can be adapted and used to identify gaps in reentry support for veterans in other states, which in turn can help those states tailor our intervention to meet their identified needs. Similarly, although our demonstration here of analyzing data through process mapping is limited to the purpose of intervention design, the visualization enabled through process mapping strongly supports the definition and clarification of shared visions that, even beyond implementation research, enable more efficient communication and collaboration among the various stakeholders involved in research efforts that require analyses of multi-perspective process data to examine health care for justice-involved populations.

## Conclusions

As interventions to improve health care for justice-involved populations continue to develop, the hope is for effective interventions to spread to numerous different care settings, and in turn to be tailored to various existing processes of care delivery. Strengths of process mapping include (i) specifying how one process is the same or different from another, (ii) detailing which exact part of the process is to be changed to make the intervention more compatible to a particular setting or population, and (iii) explaining to stakeholders, in clear terms, what the components of a program or service are and how existing barriers or problems may be addressed by a proposed change to processes. Making program processes more transparent identifies opportunities for improvement, and gains additional support for evidence-based practices, increasing the likelihood of their successful spread and uptake. Process maps can benefit discussions among researchers and justice-involved individuals, as well as among stakeholders within service-providing organizations who wish to continuously improve the delivery of services and increase positive outcomes. Our use of process mapping for understanding the reentry support process for veterans can be adapted to other implementation studies that could benefit from clear articulation of current processes and proposed changes. The visualization enabled through process mapping strongly supports the definition and clarification of a shared vision, enabling more efficient communication and collaboration among the various stakeholders involved in implementation efforts to enhance health care for justice-involved populations.

## Additional file

Additional file 1: Photograph of a process map under development through research team consensus. (PNG 578 kb)

## Abbreviations

HCRV: Health Care for Reentry Veterans
PIE: Post-Incarceration Engagement
QI: quality improvement
SIM: Sequential Intercept Model
VA: Department of Veterans Affairs
