# Supplementary figures and images for: A novel application of process mapping in a criminal justice setting to examine implementation of peer support for veterans leaving incarceration
Source: Health Justice. 2019 Mar 26;7:3. doi: 10.1186/s40352-019-0085-x (PMC6718000; doi:10.1186/s40352-019-0085-x)

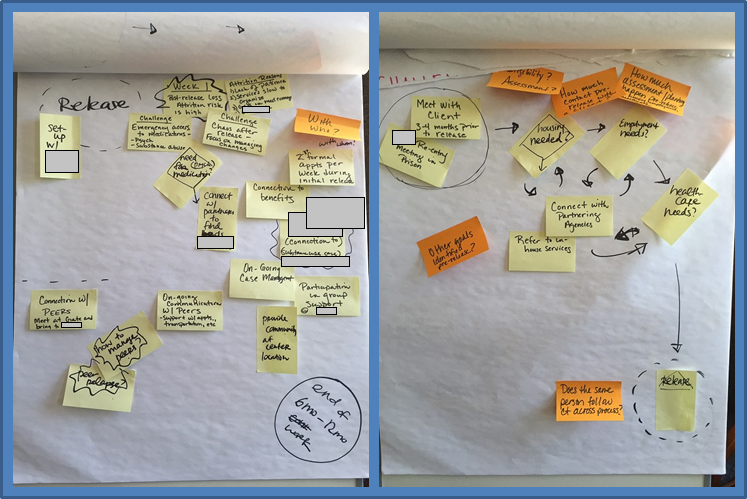

Supplement: Supplementary file 1 — Photograph of a process map under development through research team consensus. (PNG 578 kb) [file 40352_2019_85_MOESM1_ESM.png]
